# Novel Insights into Selection for Antibiotic Resistance in Complex Microbial Communities

**DOI:** 10.1128/mBio.00969-18

**Published:** 2018-07-24

**Authors:** Aimee K. Murray, Lihong Zhang, Xiaole Yin, Tong Zhang, Angus Buckling, Jason Snape, William H. Gaze

**Affiliations:** a European Centre for Environment and Human Health, University of Exeter Medical School, Environment & Sustainability Institute, Penryn, Cornwall, United Kingdom; b Department for Civil Engineering, University of Hong Kong, Hong Kong, China; c College of Life and Environmental Sciences, University of Exeter, Penryn, Cornwall, United Kingdom; d AstraZeneca Global Environment, Macclesfield, Cheshire, United Kingdom; Emory University School of Medicine

**Keywords:** antibiotic resistance, evolution, metagenomics, microbial ecology

## Abstract

Recent research has demonstrated that selection for antibiotic resistance occurs at very low antibiotic concentrations in single-species experiments, but the relevance of these findings when species are embedded in complex microbial communities is unclear. We show that the strength of selection for naturally occurring resistance alleles in a complex community remains constant from low subinhibitory to above clinically relevant concentrations. Selection increases with antibiotic concentration before reaching a plateau where selection remains constant over a 2-order-magnitude concentration range. This is likely to be due to cross protection of the susceptible bacteria in the community following rapid extracellular antibiotic degradation by the resistant population, shown experimentally through a combination of chemical quantification and bacterial growth experiments. Metagenome and 16S rRNA analyses of sewage-derived bacterial communities evolved under cefotaxime exposure show preferential enrichment for *bla*_CTX-M_ genes over all other beta-lactamase genes, as well as positive selection and co-selection for antibiotic resistant, opportunistic pathogens. These findings have far-reaching implications for our understanding of the evolution of antibiotic resistance, by challenging the long-standing assumption that selection occurs in a dose-dependent manner.

## IMPORTANCE

Antibiotic resistance is one of the greatest global issues facing society. Still, comparatively little is known about selection for resistance at very low antibiotic concentrations. We show that the strength of selection for clinically important resistance genes within a complex bacterial community can remain constant across a large antibiotic concentration range (wide selective space). Therefore, largely understudied ecological compartments could be just as important as clinical environments for selection of antibiotic resistance.

## INTRODUCTION

Antibiotic resistance poses a major threat to society, the sustainability of modern health care systems, food security, and the global economy (1, 2). Until recently, most research on evolution of resistance focused on selection at clinically relevant antibiotic concentrations, as the “traditional” selective window hypothesis was universally accepted. This hypothesis states that selection for antibiotic resistance will occur only above the MIC of susceptible bacteria and below the MIC of resistant bacteria (3). In fact, numerous experimental studies have observed selection for resistance at sub-MIC antibiotic concentrations, at the point where the selective pressure (antibiotic) is sufficient to offset the cost of resistance (3–7). In recent isogenic studies, a single host species with chromosomal or plasmid-borne resistance mechanisms was competed with its susceptible counterpart at various concentrations of antibiotic to determine the minimal selective concentration (MSC) (3, 5). The MSC is the lowest concentration of antibiotic at which resistance is positively selected, which can be significantly lower than the MIC (3, 5). MSCs have also been estimated using publicly available, clinical breakpoint data (8), but experimental data are required to assess the validity of these predictions, especially in a community context. These findings show that the selective compartment (the antibiotic gradient and spatial range along which resistant bacteria/genes could be enriched) is much larger than previously thought (9). This in turn suggests that selection may be occurring in previously unconsidered selective compartments which harbor relatively low antibiotic concentrations, such as the gut microbiome, wastewater, and even surface waters contaminated with antibiotic residues.

Though these findings are significant, the use of single species means that their relevance with regard to selection in complex microbial communities remains unclear. Many studies have quantified numbers and/or prevalence of resistance genes in wastewater influent and effluent (including, but not limited to, references 10 and 11), with a recent study utilizing emulsion, paired isolation, and concatenation PCR (epicPCR) to identify the host background of highly abundant resistance genes (12). However, positive selection for resistance within complex bacterial communities is a current knowledge gap (13), with experimental MSC data in complex communities severely lacking. One recent study (14) reported a biological effect at low concentrations of tetracycline in a microbial community, by quantifying tetracycline resistance gene prevalence (*tetA* and *tetG* genes normalized to 16S rRNA copy number). However, as starting gene frequencies were not measured, it is unclear if the observed effect was driven by positive selection or reduced negative selection. In other words, without comparing the final resistance gene prevalence to the initial resistance gene prevalence, it is unknown if resistance genes actually increased over time under tetracycline exposure (i.e., were positively selected) or if resistance genes were simply lost at a lower rate than the no-antibiotic control (i.e., were negatively selected or showed increased persistence). Here, we aimed to quantify positive selection in a complex bacterial community by conducting evolution experiments using a wastewater bacterial community inoculum to determine the MSC of cefotaxime. Co-selection for other resistance genes and effects on community structure were also determined through metagenome analyses.

Cefotaxime is a World Health Organization (WHO) recognized “critically important” antibiotic (15) “essential” for human medicine (16) that was most recently identified as a key antimicrobial stewardship target through inclusion in the WHO “watch list” of essential medicines (17). In this study, the prevalence of the *bla*_CTX-M_ gene group was determined with quantitative PCR (qPCR) and selection coefficients were calculated to estimate the MSC of cefotaxime. CTX-Ms are extended-spectrum beta-lactamases (ESBLs) which cleave the beta-lactam ring, effectively inactivating and degrading beta-lactam antibiotics (18). Previous work has demonstrated that beta-lactamases can inactivate extracellular beta-lactams, to the benefit of nearby susceptible bacteria (19–21). This protective effect on susceptible bacteria has since been shown for an intracellularly expressed resistance mechanism, also degradative in nature (22).

Results show, for the first time, that selection for *bla*_CTX-M_ genes occurs at very low, subinhibitory concentrations. We also demonstrate that selection occurs with equal potency at very low antibiotic concentrations and at concentrations greatly exceeding those used in the clinic. Therefore, antibiotic resistance is not always selected for in a dose-dependent manner. These findings illustrate the importance of studying selection for resistance within complex bacterial communities over a wide selective range, representative of different selective compartments (9).

## RESULTS

### Cefotaxime exposure affects community structure.

Complex community (raw, untreated wastewater) microcosms were spiked with a range of cefotaxime concentrations. The exposure concentration range was selected from the EUCAST (23) defined clinical breakpoint concentration (the concentration at which *Enterobacteriaceae* are considered “clinically” resistant) down to 0, in a 2-fold dilution series including concentrations similar to those previously measured in different environments (such as hospital effluent, wastewater treatment plant effluent, and surface waters [few micrograms/liter up to 150 µg/liter {24–26}]). Bacterial communities were transferred daily into fresh medium and fresh antibiotic for 8 days. Chemical quantification was performed at the beginning of day 0 and after 24 h, to determine an accurate MSC (based on measured rather than nominal antibiotic concentration) and to assess the chemical stability of cefotaxime (a third-generation cephalosporin of the beta-lactam class of antibiotics) in the system.

At the end of the experiment, three replicates from a low, medium, and high antibiotic concentration were selected to undergo metagenome analyses alongside the unexposed control to identify the key resistance genes under selection. Metagenome DNA was sequenced with an Illumina MiSeq2 sequencer.

**FIG 1:**
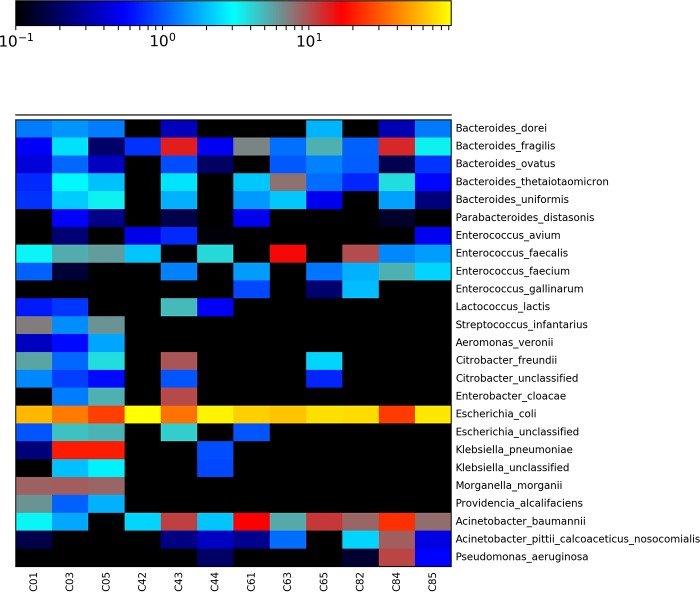
Heat map showing the relative abundance of detected species using Bray-Curtis distance measurements for treatment (*x* axis) and species (*y* axis) for each cefotaxime treatment. “C0,” “C4,” “C6,” and “C8” correspond to 0, 125, and 500 μg/liter and 2 mg/liter cefotaxime, respectively. The number after the concentration denotes the biological replicate number (1 to 5), chosen randomly for sequencing at day 8 of the experiment.

16S rRNA data were extracted from trimmed, quality-controlled paired reads with MetaPhlAn2 (27). Overall, the community comprised predominantly Gram-negative bacteria, though Gram-positive bacteria were also detected (see Fig. S1 in the supplemental material). Between-sample variation was expected due to unavoidable heterogeneity within the complex community inoculum. Even so, there were clear differences between the control untreated bacterial community and the communities exposed to cefotaxime among the 25 most abundant species (Fig. 1; all detected species can be seen in Fig. S1). In particular, several species were eliminated by cefotaxime treatment (or reduced below the limit of detection). These included the opportunistic Gram-negative pathogens Providencia alcalifaciens, Aeromonas veronii, Morganella morganii, and Klebsiella pneumoniae, as well as the opportunistic Gram-positive pathogen Streptococcus infantarius. All of these were significantly associated with the no-antibiotic control as determined by linear discriminant analysis (LDA) effect size (LEfSe) analyses (Fig. S2). Conversely, several Gram-negative and Gram-positive opportunistic pathogens showed greater abundance in treated communities than in the untreated control, namely, Pseudomonas aeruginosa, Acinetobacter baumannii, Bacteroides fragilis, and Enterococcus faecalis; however, only P. aeruginosa was significantly enriched in the 2 mg/liter cefotaxime treatment (LEfSe, Fig. S2). Cefotaxime treatment also resulted in slightly decreased numbers of Escherichia coli, though this was not significant and it was still the predominant species across all treatments. In general, there was much greater variability between treatment replicates compared to the untreated control (Fig. S1).

10.1128/mBio.00969-18.1 FIG S1  Heat map showing relative abundance of all detected species using Bray-Curtis distance measurements for treatment (*x* axis) and species (*y* axis) for each cefotaxime treatment. “C0,” “C4,” “C6,” and “C8” correspond to 0, 125, 500, and 2,000 μg/liter cefotaxime, respectively. The number after the concentration denotes the biological replicate number (1 to 5), chosen randomly for sequencing at day 8 of the experiment. Download FIG S1, DOCX file, 0.3 MB. Copyright © 2018 Murray et al. 2018 Murray et al. This content is distributed under the terms of the Creative Commons Attribution 4.0 International license.

10.1128/mBio.00969-18.2 FIG S2  Linear discriminant analysis (LDA) effect size (LEfSe) analyses of statistically significant species associated with different cefotaxime treatments. Negative LDA scores (red) show species enriched in the no-antibiotic treatment, and positive LDA scores (green) show species enriched in the 2,000 µg/liter cefotaxime treatment. Download FIG S2, DOCX file, 0.1 MB. Copyright © 2018 Murray et al. 2018 Murray et al. This content is distributed under the terms of the Creative Commons Attribution 4.0 International license.

### *bla*_CTX-M_ genes are preferentially selected over all other beta-lactam resistance mechanisms.

Metagenome data were further analyzed with the ARGs-OAP pipeline (28), designed to thoroughly interrogate metagenome data and identify resistance genes. Selection for beta-lactam resistance was prominent (as expected); however, co-selection for resistance to unrelated antibiotic classes was also observed, namely, co-selection for resistance to macrolides, aminoglycosides, trimethoprim, tetracyclines, and sulfonamides, which is likely to be due to carriage of multiresistance plasmids (see Fig. S3).

10.1128/mBio.00969-18.3 FIG S3  Heat map showing average (biological replicate, *n* = 3) resistance gene relative abundance (resistance gene number normalized with 16S rRNA copy number), following 8 days of culture with cefotaxime. Macrolide-Linco-Strept, macrolide, lincosamide, streptogramin resistance genes. Download FIG S3, DOCX file, 0.1 MB. Copyright © 2018 Murray et al. 2018 Murray et al. This content is distributed under the terms of the Creative Commons Attribution 4.0 International license.

**FIG 2:**
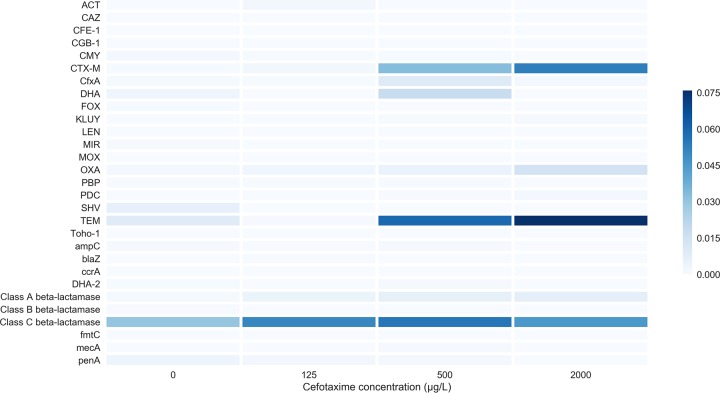
Heat map showing average (*n* = 3) detected beta-lactam resistance gene subtype relative abundance (resistance gene number normalized with 16S rRNA copy number), following 8 days of culture with cefotaxime. Only genes detected with the ARGs-OAP pipeline are shown.

We delved deeper into the beta-lactam resistance genes to determine which genes, if any, were preferentially selected. We observed substantial enrichment for the beta-lactamase and extended-spectrum beta-lactamase (ESBL) genes *bla*_TEM_, *bla*_OXA_, and *bla*_CTX-M_ (Fig. 2). Average increases in relative abundance from the lowest to highest concentration were 8-fold, 8-fold, and 70-fold, respectively. *bla*_CTX-M_ was preferentially selected over all other beta-lactamase-encoding genes at each cefotaxime concentration.

### The MSC of cefotaxime is very low, but selection plateaus across a large concentration range.

Given the strong positive selection for *bla*_CTX-M_, we focused on accurate quantification of this group of genes across the entire experimental antibiotic gradient using qPCR. This follows previous work which showed that qPCR is the most sensitive method for MSC determination (14). *bla*_CTX-M_ gene copy number was normalized to 16S rRNA copy number, to determine a molecular prevalence of *bla*_CTX-M_; this prevalence was determined for each cefotaxime concentration at both the beginning and end of the experiment. A Kruskal-Wallis test confirmed that *bla*_CTX-M_ prevalence, 16S rRNA copy number, and *bla*_CTX-M_ copy number (all *n* = 5 each) did not differ significantly between treatments at day 0.

**FIG 3:**
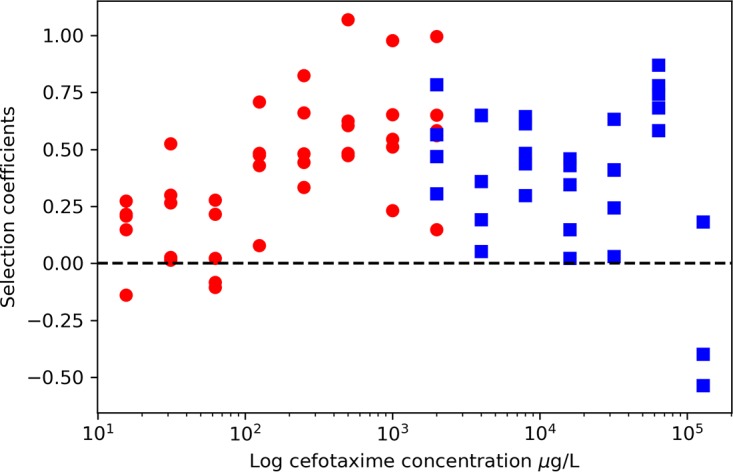
Selection coefficients (*n* = 5) for each cefotaxime concentration, which equal the natural log of resistance gene prevalence (*bla*_CTX-M_ gene/16S rRNA copy number) at day 0 and day 8. Circles, selection coefficients from low-concentration experiment; squares, selection coefficients from high-concentration experiment. Selection coefficients of >0 indicate positive selection.

Selection coefficients based on change in *bla*_CTX-M_ prevalence over time were calculated and plotted against cefotaxime concentration as in previous single-species assays (3, 5) (Fig. 3). A positive selection coefficient value indicates that positive selection is occurring, and the *x* axis intercept estimates the MSC, here 0.4 µg/liter (Fig. S4).

10.1128/mBio.00969-18.4 FIG S4  MSC (cefotaxime concentration at the *x* axis intercept) determination using average (*n* = 5) selection coefficients (natural log of *bla*_CTX-M_ prevalence over 8 days, *bla*_CTX-M_ prevalence = *bla*_CTX-M_ copy number/16S rRNA copy number; qPCR technical replicate, *n* = 2). Shown with standard error bars (of biological replicates) and polynomial (order 2) line of best fit. Download FIG S4, DOCX file, 0.04 MB. Copyright © 2018 Murray et al. 2018 Murray et al. This content is distributed under the terms of the Creative Commons Attribution 4.0 International license.

*bla*_CTX-M_ prevalence increased over time (Fig. S5) and with antibiotic concentration (linear term: *F*_1_, 42 = 26.3, *P* < 0.001) but appeared to plateau at 500 µg/liter (quadratic term: F_1_, 42 = 13.2, *P* < 0.001), and so an additional experiment was performed to determine if this plateau continued at higher concentrations (Fig. 3). As hypothesized, *bla*_CTX-M_ prevalence increased when exposed to cefotaxime (linear term: F_1_, 36 = 9.6, *P* < 0.01) but remained relatively constant (quadratic term: F_1_, 36 = 9.4, *P* < 0.01) up until the two highest concentrations used in this study (Fig. 3). These concentrations are over 30 times and 50 times the defined clinical breakpoint cefotaxime concentration of 2 mg/liter for *Enterobacteriaceae*. The rise in *bla*_CTX-M_ prevalence at 64 mg/liter was due to an increase in *bla*_CTX-M_ gene copy number, and the decrease at 128 mg/liter was due to a significant decrease in *bla*_CTX-M_ and slight reduction in 16S rRNA copy number (Fig. S6 and S7).

10.1128/mBio.00969-18.5 FIG S5  Average (biological replicate, *n* = 5l technical qPCR replicate of each biological replicate, *n* = 2) *bla*_CTX-M_ prevalence (*bla*_CTX-M_ copy number/16S rRNA copy number) at day 0 and following 1, 4, and 8 days of cefotaxime exposure. Shown with standard error bars (of biological replicates). Download FIG S5, DOCX file, 0.04 MB. Copyright © 2018 Murray et al. 2018 Murray et al. This content is distributed under the terms of the Creative Commons Attribution 4.0 International license.

10.1128/mBio.00969-18.6 FIG S6  Average (biological replicate, *n* = 5; technical qPCR replicate of each biological replicate, *n* = 2) *bla*_CTX-M_ copy number following 8 days of cefotaxime exposure in the higher-concentration experiment. Shown with standard error bars (of biological replicates). Download FIG S6, DOCX file, 0.04 MB. Copyright © 2018 Murray et al. 2018 Murray et al. This content is distributed under the terms of the Creative Commons Attribution 4.0 International license.

10.1128/mBio.00969-18.7 FIG S7  Average (biological replicate, *n* = 5; technical qPCR replicate of each biological replicate, *n* = 2) 16S rRNA copy number following 8 days of cefotaxime exposure, in the higher-concentration experiment. Shown with standard error bars (of biological replicates). Download FIG S7, DOCX file, 0.05 MB. Copyright © 2018 Murray et al. 2018 Murray et al. This content is distributed under the terms of the Creative Commons Attribution 4.0 International license.

### The bacterial community readily degrades cefotaxime.

**FIG 4:**
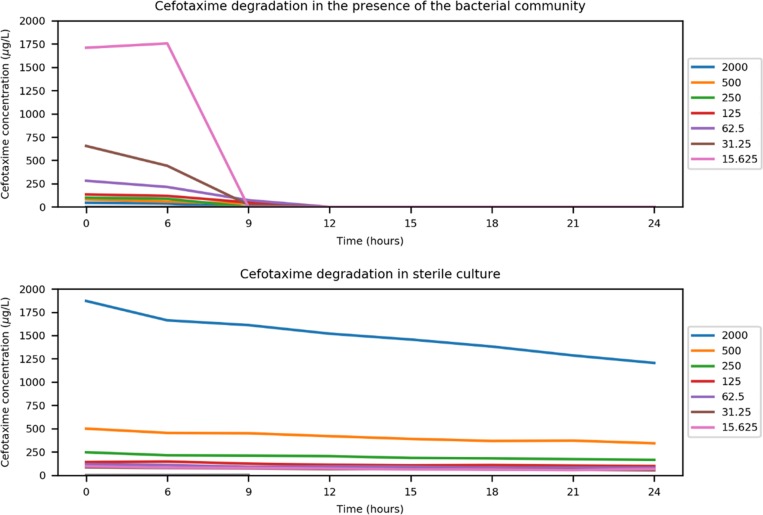
Single biological replicate and duplicate chemical replicate quantification of cefotaxime (measured cefotaxime concentration µg/liter) at 0 and 6 h and then every 3 h for 24 h at different starting cefotaxime concentrations (micrograms/liter) in the presence of the complex bacterial community and in sterile culture.

We hypothesized that this plateau in selection was due to both the mechanism and sociality of the *bla*_CTX-M_ genes: as beta-lactamase enzymes can be found both intracellularly and extracellularly (21), the plateau in *bla*_CTX-M_ prevalence may be due to negative frequency-dependent selection (19). In other words, the more prevalent *bla*_CTX-M_ becomes, the lower its fitness as cefotaxime degradation is accelerated to the benefit of the entire community, including non-*bla*_CTX-M_-bearing competitors. To investigate if cefotaxime was degraded by the community, chemical quantification of cefotaxime in the presence of the community was performed. Incubating the microcosms for 24 h resulted in complete degradation of cefotaxime, at all but the highest concentration (Table S1). All measured concentrations were lower than expected, and the lowest concentration (15.625 µg/liter) was below the limit of detection at the beginning of the assay. Therefore, the MSC (0.4 µg/liter) is estimated based on nominal concentrations but in reality is likely to be lower still. As cefotaxime is known to be relatively unstable (29), an overnight degradation experiment was conducted to determine the amount of biotic and abiotic degradation occurring in the experimental system. A sterile microcosm and another inoculated with the complex community were incubated and destructively sampled at 0 h, 6 h, and then every 3 h for 24 h. In sterile culture, cefotaxime had only partially degraded over 24 h, whereas in the presence of the community, cefotaxime was undetectable following 12 h of incubation (Fig. 4). This increase in degradation rate coincided with the beginning of the exponential growth phase of the community (Fig. S8).

10.1128/mBio.00969-18.8 FIG S8  Growth (optical density at 600 nm) of the complex community over time during the 24-h degradation experiment. Single replicate only. Download FIG S8, DOCX file, 0.1 MB. Copyright © 2018 Murray et al. 2018 Murray et al. This content is distributed under the terms of the Creative Commons Attribution 4.0 International license.

10.1128/mBio.00969-18.9 TABLE S1  Nominal (expected) and average (biological replicate, *n* = 3; technical replicate of each *n* = 2) measured cefotaxime concentrations as determined by liquid chromatography-mass spectrometry at the beginning (time zero) of the selection experiment and after 24 h of culture at 180 rpm, 37°C, in the presence of the complex community. Also shown are the cefotaxime stocks (“1” and “2”) used in the experiment. Download TABLE S1, DOCX file, 0.01 MB. Copyright © 2018 Murray et al. 2018 Murray et al. This content is distributed under the terms of the Creative Commons Attribution 4.0 International license.

### Extracellular beta-lactamases “protect” susceptible bacteria at cefotaxime concentrations well above the MIC.

**FIG 5:**
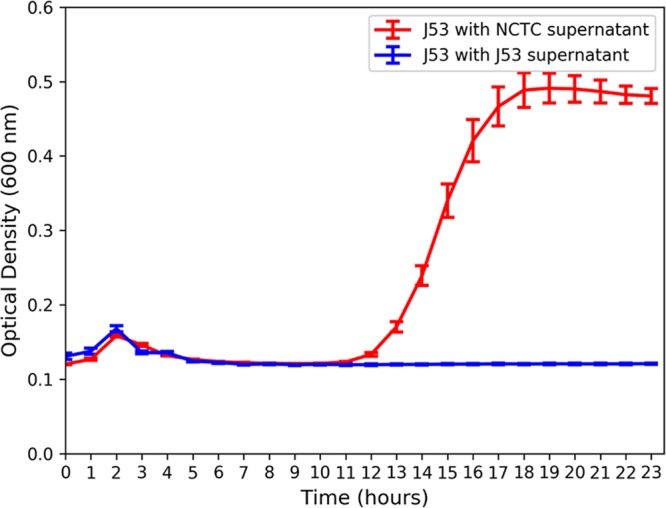
Average (*n* = 4) optical density (600 nm) over time of susceptible E. coli strain J53 grown in 2 mg/liter cefotaxime (clinical breakpoint concentration for *Enterobacteriaceae*) with beta-lactamase-containing supernatant (NCTC strain 13451) or beta-lactamase-free supernatant (strain J53).

To confirm that this degradation was biotic and by extracellular beta-lactamases, an additional experiment was performed whereby a susceptible E. coli strain (J53) was cultured in the presence of supernatant derived from an overnight culture of an E. coli strain bearing *bla*_CTX-M_-_15_, *bla*_TEM-1_, and *bla*_OXA-1_ on a fully sequenced resistance plasmid (30) (strain NCTC 13451, available from Public Health England). Addition of the supernatant from the resistant strain allowed growth of the susceptible strain at the clinical breakpoint concentration (23), which was over 10 times the MIC of the susceptible strain (Fig. 5).

## DISCUSSION

Here, we quantified positive selection for antibiotic (cefotaxime) resistance in a wastewater-derived complex bacterial community, by quantifying changes in resistance gene prevalence over time. We show that clinically important (31) resistance genes (*bla*_CTX-M_) were positively selected at very low, environmentally relevant concentrations likely due to a combination of clonal expansion of hosts carrying *bla*_CTX-M_ and horizontal gene transfer of plasmids bearing *bla*-_CTX-M_. Antibiotic quantification has been identified as an overlooked aspect of MSC determination (13). We quantified antibiotic concentrations when determining the MSC and found cefotaxime to be rapidly degraded by the community, suggesting the estimated MSC of 0.4 µg/liter to be an underestimate. Even so, the cefotaxime MSC determined in this study was very similar to several measured environmental concentrations (25, 32), suggesting that selection could occur in certain environments such as hospital effluent and wastewater influent. Responses to selection in such environments may be reduced, as unenriched bacterial communities, e.g., in sewage, may be impacted by high cell numbers and associated reduction in resource availability. However, it is also possible that sustained exposure over long periods would produce the same response.

In addition, we observed a plateau in the strength of selection across a very large antibiotic concentration range. This novel finding contrasts with previous work which has shown resistance to increase monotonically with antibiotic concentration (3, 5, 14). A crucial implication of this finding is that selection for clinically important resistance mechanisms, such as *bla*_CTX-M_, may occur to a similar extent at subinhibitory concentrations as at high, clinical concentrations. The observed plateau in resistance selection has clinical relevance, when considering the antibiotic concentration gradients which inevitably form in different body compartments during chemotherapy (33). These may also provide greater potential for selection for antibiotic resistance *in vivo* than previously considered. Potential overtreatment with unnecessarily long antibiotic courses (34) may compound this effect. Future research should address this finding and its relevance to environmental protection, effective antibiotic treatment, and antimicrobial stewardship.

The observed plateau in selection for resistance is likely due to the cross-protective effect conferred by the resistant fraction on the susceptible fraction of bacteria in the population. Three lines of evidence strongly support this: (i) the degradative effect of the community, whereby within 24 h all cefotaxime is degraded below the limit of chemical quantification, including the very highest, clinical breakpoint concentration; (ii) metagenome analyses of 3 replicates at 4 antibiotic concentrations, which showed that the main mechanism of resistance to the treatment antibiotic was degradative in nature and could therefore provide a benefit to susceptible competitors within the community; (iii) the single-species E. coli experiment, which used supernatant from a resistant strain bearing a multiresistance plasmid with the plasmid-free strain to show the potential extent of this community-wide benefit. The level of protection conferred by extracellular beta-lactamases in the supernatant of the resistant strain culture allowed growth of the susceptible strain well above its own MIC, at the clinical breakpoint concentration. Extrapolating this finding to the community, we hypothesize that within each 24 h period, CTX-M producers (and possibly other degraders) are selected for by cefotaxime and are then outcompeted by susceptible bacteria following antibiotic degradation. This means that resistant genotypes are likely to persist at even very low antibiotic concentrations, as they provide a benefit to the whole community; this effect has been modeled previously (19).

Selection for *bla*_CTX-M_ genes is likely due to a combination of clonal expansion of hosts carrying *bla*_CTX-M_ and horizontal gene transfer of plasmids bearing *bla*-_CTX-M_. Our results are consistent with epidemiological data on beta-lactam resistance genes (35, 36), which document the rapid spread of *bla*_CTX-M_ genes worldwide to a “pandemic” status. In this study, *bla*_CTX-M_ genes were under stronger selection than a large diversity of other resistance genes, possibly due to lower fitness cost (either metabolic or due to genetic context) and/or due to more efficient degradation and a potential wider degradative capacity. For example, previous research found that the MICs of *bla*_CTX-M_-positive bacteria isolated from river sediment downstream of a wastewater treatment plant were in excess of 2,048 µg/ml (37). Additionally, many TEM and OXA beta-lactamases do not have the extended-spectrum degradative capability of CTX-M ESBLs (35, 36). We suggest that the ability of *bla*_CTX-M_ genes to outcompete other beta-lactamase genes at all studied concentrations may also have contributed to the pandemic spread of *bla*_CTX-M_ genes worldwide (35) and the replacement of other beta-lactamase variants (36).

The metagenome analyses showed that *ampC* genes were detected but not enriched by cefotaxime exposure. Overexpression of chromosomal *ampC* genes can increase levels of resistance to many antibiotics, including cefotaxime, but these genes were very rare within metagenomes and confer only low-level resistance up to 8 mg/liter (38), suggesting that they do not play a significant role at the community level in this study (39). The metagenome analyses also showed that cefotaxime can also co-select for resistance to a range of antibiotic classes, even at subinhibitory cefotaxime concentrations. Co-occurrence of *bla*_CTX-M_ genes and aminoglycoside resistance has been reported previously and is confirmed here (36). High levels of co-selection also indicate the presence of multidrug resistant plasmids, via co-resistance (i.e., co-localization of multiple genes, of which only one needs to be under positive selection) (40).

The bacterial community analyses showed that cefotaxime had significant effects on community structure, even at subinhibitory concentrations, through elimination of several species as determined by LEfSe. The bacterial communities in all treatments comprised mainly of Gram-negative bacterial families, with E. coli being the most abundant species in all treatments. In wastewater, with a lower temperature and different nutritional composition, interspecies competition with other taxa could result in reduced E. coli abundance, which may be favored by the medium and temperature used in this study. However, E. coli is known to survive wastewater treatment and is used as a fecal indicator organism to assess water quality (41). Therefore, in wastewater, E. coli may still possess a competitive advantage. There was much greater variability between replicates (for all species other than E. coli) within exposed communities, indicative of potential founder effects on evolution within individual microcosms, again supporting the hypothesis that selection was acting at these concentrations. Worryingly, cefotaxime exposure enriched for well-known opportunistic Gram-negative pathogens, including P. aeruginosa, which readily infects immunocompromised individuals such as cystic fibrosis patients, and A. baumannii, which is most commonly associated with hospital-acquired infections (42). Enrichment for these opportunistic pathogens and human and gut commensals such as B. fragilis and E. faecalis under cefotaxime exposure may be due to intrinsic resistance, which is likely to result in enrichment within communities including susceptible strains.

In summary, our findings develop understanding of selection for antibiotic resistance by showing that the strength of selection for clinically important resistance genes in a community context can be equal across a very large antibiotic concentration gradient; in other words, we show that the strength of selection within a given selective space may be constant. Therefore, selection pressure below the MIC of susceptible bacteria may be as strong as selection between the MICs of susceptible and resistant bacteria (traditional selective window). We hypothesize that in our study, this observation is due to the community-wide benefit provided by resistant bacteria harboring degradative resistance mechanisms. Marked increases in common Gram-negative, opportunistic pathogens and co-selection for resistance to other antibiotic classes raise concerns about selection and co-selection for clinically relevant genes (such as *bla*_CTX-M_) in pathogenic hosts occurring in a wide range of ecologic compartments.

## MATERIALS AND METHODS

### Complex community collection, storage, and preparation.

Domestic sewage influent from a wastewater treatment plant serving a small town was collected in October 2015. The treatment plant serves a population of 43,000. Single-use aliquots were mixed in a 1:1 ratio with 20% glycerol, vortexed, and stored at −80°C. Before use, samples were spun down at 21,100 × *g* for 10 min, the supernatant was removed, and the pellet was resuspended twice in equal volumes of 0.85% NaCl to prevent nutrient/chemical carryover.

A pilot experiment (data not shown) was conducted to determine the appropriate density of the complex community inoculum by comparing growth over 24 h of different dilutions of inoculum at a range of antibiotic concentrations in a 96-well plate. Following results from the pilot experiment, the 10% (vol/vol) wastewater inoculum was used for all further experiments using the complex community on the basis that it produced the tightest replicates at all the time points and the most reliable growth phases over 24 h.

### Selection experiments.

Iso-Sensitest broth (Sigma) was inoculated with 10% (vol/vol) of untreated wastewater. This was separated into 30-ml aliquots, and appropriate amounts of cefotaxime stock solution were added. Cefotaxime (Molekular) stocks were prepared in autoclaved and filtered (0.22-µm) deionized water.

These 30-ml aliquots were further separated into 5-ml aliquots, with 5 replicates for each of the cefotaxime assay concentrations: 2 mg/liter, 1 mg/liter, and 500, 250, 125, 62.5, 31.25, 15.625, and 0 µg/liter for the first experiment and 128, 64, 32, 16, 8, 4, 2, and 0 mg/liter for the second experiment.

All replicates were immediately sampled for the day 0 sampling time point: 2 portions of 1 ml of each replicate for each treatment were spun down at 21,100 × *g* for 3 min, the supernatant was removed, and pellet was resuspended in 1,000 µl 20% glycerol followed by storage at −80°C. All other samples for DNA extraction were taken following each overnight incubation at 37°C, with 180-rpm shaking, as described above.

After each incubation, 50 µl of each microcosm was inoculated into 5 ml fresh medium with fresh antibiotic, and samples were taken as described above for a total of 8 days. Remaining cell suspensions were spun down and stored as described above at the end of each experiment.

### Metagenome analyses.

Three replicates were chosen at random from the no-antibiotic, 125 µg/liter, 500 µg/liter, and 2 mg/liter treatments to undergo shotgun metagenome sequencing on the MiSeq2 v2 platform at University of Exeter Sequencing Service (ESS).

DNA was extracted from 1 ml of frozen overnight culture using the Mo Bio extraction kit according to the manufacturer’s instructions. DNA was cleaned and concentrated using AMPure beads. First, 2 µl of 20 mg/ml RNase A (Qiagen) was added to 50 µl DNA and incubated for 10 min at 37°C. Fifty microliters of AMPure beads was mixed with the DNA/RNase solution, mixed gently by pipetting, and incubated at room temperature for 5 to 10 min. Following pulse centrifugation to collect droplets, tubes were placed on a magnetic stand and left until all beads had precipitated to the side of the tube. Supernatant was removed, and beads were washed two times with 300 µl freshly prepared 80% ethanol. Beads were air dried briefly (1 to 2 min), resuspended in 10 µl 10 mM Tris-HCl, and then incubated for another 10 min at 50°C. Following pulse centrifugation and bead precipitation, DNA was transferred into a fresh tube and stored at −20°C until library preparation and sequencing.

The 12 Nextera library preparations, quality control, sequencing, and primary sequencing analysis (including trimming reads of the barcodes) were performed by ESS. Data were then run through the ARGs-OAP (online analysis pipeline for antibiotic resistance genes detection from metagenome data using an integrated structured antibiotic resistance gene database) (28). This provides the abundance of different resistance gene classes and subtypes within these groups normalized by parts per million, 16S rRNA copy number, and cell number. For all subsequent analysis, data normalized by 16S rRNA copy number were used to be in accordance with the qPCR data generated. Heat maps for resistance gene abundance were generated using pandas (43), Matplotlib (44), and Seaborn (45) Python packages for resistance gene class and beta-lactam resistance gene subtype, for averages from these three replicates.

16S rRNA sequences were extracted from the Illumina sequencing data as follows: first, forward and reverse reads were adapter trimmed using Skewer (46) in paired-end mode. FastQC (47) and MultiQC (48) verified successful adapter removal and that sequences were of acceptable quality before paired-end reads were combined with FLASH version 2 (49) with the maximum overlap set to 300. 16S rRNA sequences were extracted and assigned to bacterial species using MetaPhlAn2 (27). The resulting heat map of species relative abundance was generated using HClust2 using Bray-Curtis distance measurements between samples and features (species) (50), and overall species abundance across treatments was visualized with GraPhlAn (51). Linear discriminant analysis (LDA) effect size (LEfSe) analyses were performed, and results were visualized with LEfSe (52) to identify which species, if any, were enriched by a particular cefotaxime treatment.

### qPCR analyses.

Frozen samples were thawed, and DNA was extracted using the MBio UltraClean DNA extraction kit according to instructions. DNA was diluted 5× to 10× before use.

The qPCR conditions were optimized using primers from previous studies (53, 54): 10 µl Brilliant qPCR SYBR green master mix, 2 µl primer pair (5 µM [each] forward and reverse primers for 16S rRNA, and 9 µM [each] forward and reverse CTX-M primers), 0.2 µl bovine serum albumin (BSA) (20 mg/ml), 0.4 µl ROX reference dye (20 µM), 5 µl diluted DNA template, and filtered, sterilized water to a total volume of 20 µl. The qPCR program for all reactions was 95°C for 3 min, followed by 40 cycles of 95°C for 10 s and 60°C for 30 s. *bla*_CTX-M_ copy number was divided by 16S rRNA copy number to determine *bla*_CTX-M_ gene/16S rRNA, a proxy for *bla*_CTX-M_ prevalence.

gBlock synthetic genes (Integrated DNA Technologies [Table 1]) were used as qPCR standards; these were resuspended in Tris-EDTA (TE) buffer according to the manufacturer’s instructions and were stored at −80°C.

**TABLE 1:** List of synthetic gene blocks used in the study, their DNA sequence, size in base pairs, and accession numbers used to for design

Gene	Sequence	Size (bp)	Accession no.
16S	ACGGTGAATACGTTCCCGGGCCTTGTACACACCGCCCGTCACACCATGGGAGTG GGTTGCAAAAGAAGTAGGTAGCTTAACCTTCGGGAGGGCGCTTACCACTTTGTG ATTCATGACTGGGGTGAAGTCGTAACAAGGTAACCG	144	CP018770.2
CTX-M	GATGTGCAGCACCAGTAAAGTGATGGCCGCGGCCGCGGTGCTGAAGAAAAGTGA AAGCGAACCGAATCTGTTAAATCAGCGAGTTGAGATCAAAAAATCTGACCTTGTTAA CTATAATCCGATTGCGGAAAAGCACGTCAATGGGACGATGTCACTGGCTGAGCTTA GCGCGGCCGCGCTACAGTACAGCGATAACGTGGCGATGAATAAGCTGATTGCTCACG TTGGCGGCCCGGCTAGCGTCACCGCGTTCGCCCGACAGCTGGGAGACGAAACGTTCC GTCTCGACCGTACCGAGCCGACGTTAAACACCGCCATTCCGGGCGATCCGCGTGATA	338	KX452391.1

Standards were 10× serially diluted in TE buffer and stored at −20°C before use. Every PCR plate was always run with 5 serial dilutions of standards in duplicate (and a duplicate negative control). Provided that the efficiency for the reaction was between 90% and 110%, the average threshold cycle (*C*_*T*_) for the duplicate technical replicates for each sample was used to calculate the copy number based on a “gold standard” standard series, where the DNA concentration had been quantified by Qubit and the copy number per microliter quantified immediately prior to cycling.

### Data analyses.

All statistics were performed in RStudio (55). Selection coefficients were used to determine MSC, with the following equation, as described previously (3): {ln[*R*(*t*)/*R*(0)]}/(*t*), where *R* is resistance prevalence, *t* is time in days, and *R*(0) is resistance prevalence at time zero. The MSC is estimated by the line of best-fit *x*-axis intersect. Figures were generated with various Python packages (43–45).

### Chemical extraction/analyses.

Complex community microcosms were sampled at the day 0 point and after the first 24 h. Antibiotic stocks were also quantified.

The extraction procedure was as follows: 400 µl of culture was mixed with 400 µl HiPerChromosolv acetonitrile in a 2-ml 96-well plate and spun at 3,500 rpm for 30 min. One hundred microliters of this supernatant was then mixed with 900 µl of a 1:4 ratio of acetonitrile to high-pressure liquid chromatography (HPLC)-grade water in a fresh plate and stored in the refrigerator. Antibiotic stocks were diluted to a final concentration of 100 ng/liter in 1:4 acetonitrile. Extraction mixtures were kept at 4°C until processing.

Each concentration from the evolution experiment had a minimum of two chemical replicates from at least two of the biological replicates. Stocks were single replicate only.

### Antibiotic degradation experiment.

Washed, untreated wastewater was diluted in 25-ml Iso-Sensitest broth aliquots spiked with cefotaxime concentrations of 0, 15.625, 31.25, 62.5, 125, 250, and 500 µg/liter or 2 mg/liter. A sterile control at the same concentrations was also prepared. These were incubated at 37°C, with 180-rpm shaking in between samplings. Chemical extractions (see below) and destructive sampling for optical density (OD) readings were performed at time zero and then every 3 h for 24 h. OD measurements were carried out in a spectrophotometer (Jenway, United Kingdom) at the same time points at 600 nm. Any samples with OD readings of a value greater than 1 were diluted 10× in Iso-Sensitest broth and then remeasured.

### Supernatant experiment.

E. coli strains J53 and NCTC 13451 were grown overnight at 37°C with shaking at 180 rpm in Iso-Sensitest broth (supplemented with 2 mg/liter cefotaxime for NCTC 13451). This concentration was chosen on the basis that it was greater than the J53 MIC (>250 µg/liter and <500 µg/liter, determined by microdilution assay [56]), that it would be fully degraded in a beta-lactamase-producing community (according to the overnight degradation experiment), and because it is the clinical breakpoint concentration for *Enterobacteriaceae* (23). The supernatants from both overnight cultures were spun down at 21,000 × *g* for 2 min twice and then filtered through a 0.22-µm filter. J53 was inoculated at a starting optical density (600 nm) of 0.01 into fresh Iso-Sensitest broth amended with 2 mg/liter and J53 or NCTC 13451 supernatant (12.5% [vol/vol]). Controls included a blank control (to check general aseptic technique), broth with each supernatant (to verify that the supernatant was sterile), and J53 in broth both with and without antibiotic (to deduce effects of nutrient dilution).
