# Supplementary material for: Novel Insights into Selection for Antibiotic Resistance in Complex Microbial Communities
Source: mBio. 2018 Jul 24;9(4):e00969-18. doi: 10.1128/mBio.00969-18 (PMC6058293; doi:10.1128/mBio.00969-18)
Supplement: FIG S1 [file mbo004183973sf1.docx]

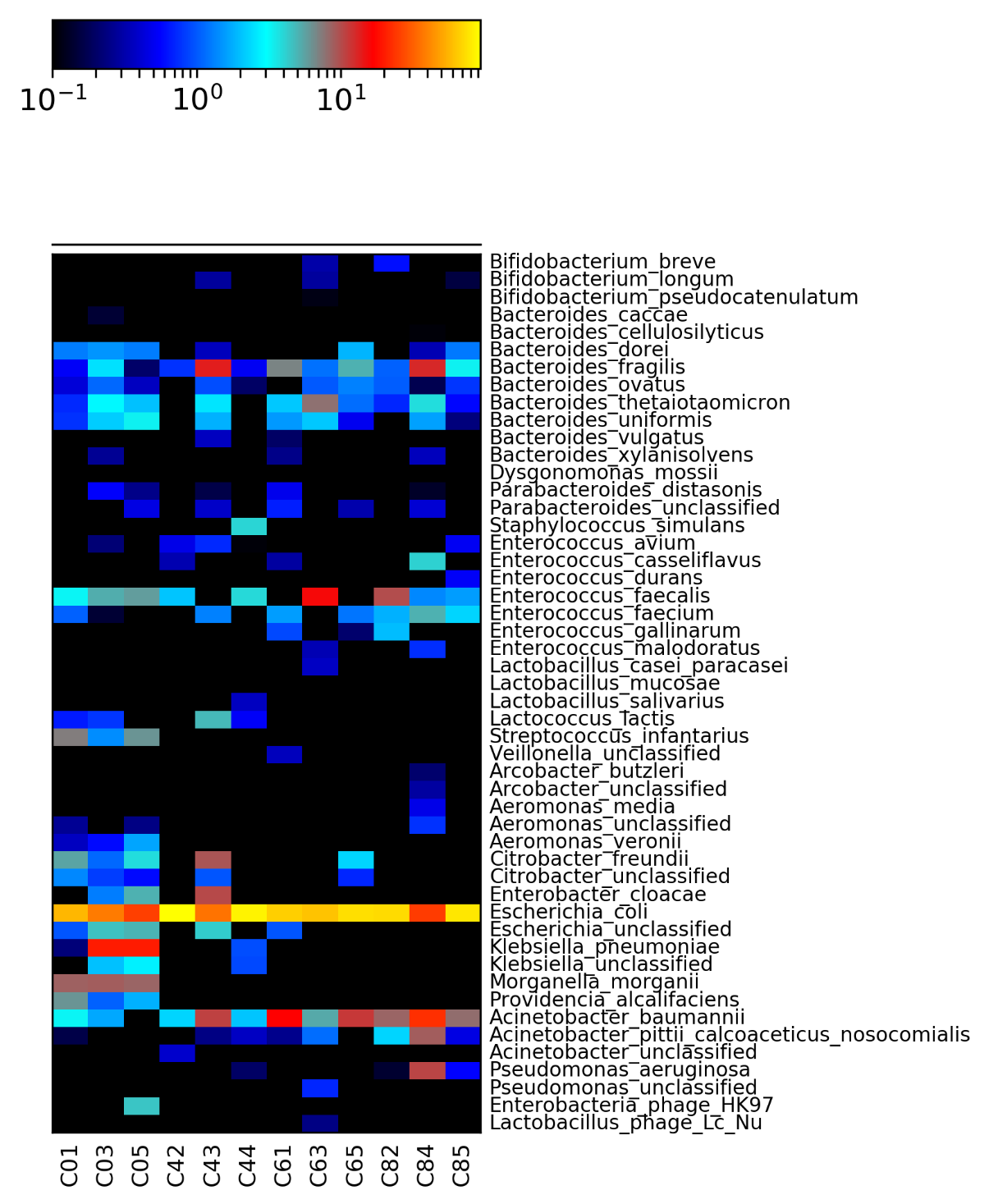


Figure S1. Heatmap showing relative abundance of all detected species using Bray Curtis distance measurements for treatment (x axis) and species (y axis) for each cefotaxime treatment. ‘C0’, ‘C4’, ‘C6’ and ‘C8’ correspond to 0, 125, 500 and 2000 µg/L cefotaxime respectively. The number after the concentration denotes the biological replicate number (1 – 5), chosen randomly for sequencing at day 8 of the experiment.
