# Supplementary material for: Novel Insights into Selection for Antibiotic Resistance in Complex Microbial Communities
Source: mBio. 2018 Jul 24;9(4):e00969-18. doi: 10.1128/mBio.00969-18 (PMC6058293; doi:10.1128/mBio.00969-18)
Supplement: FIG S2 [file mbo004183973sf2.docx]

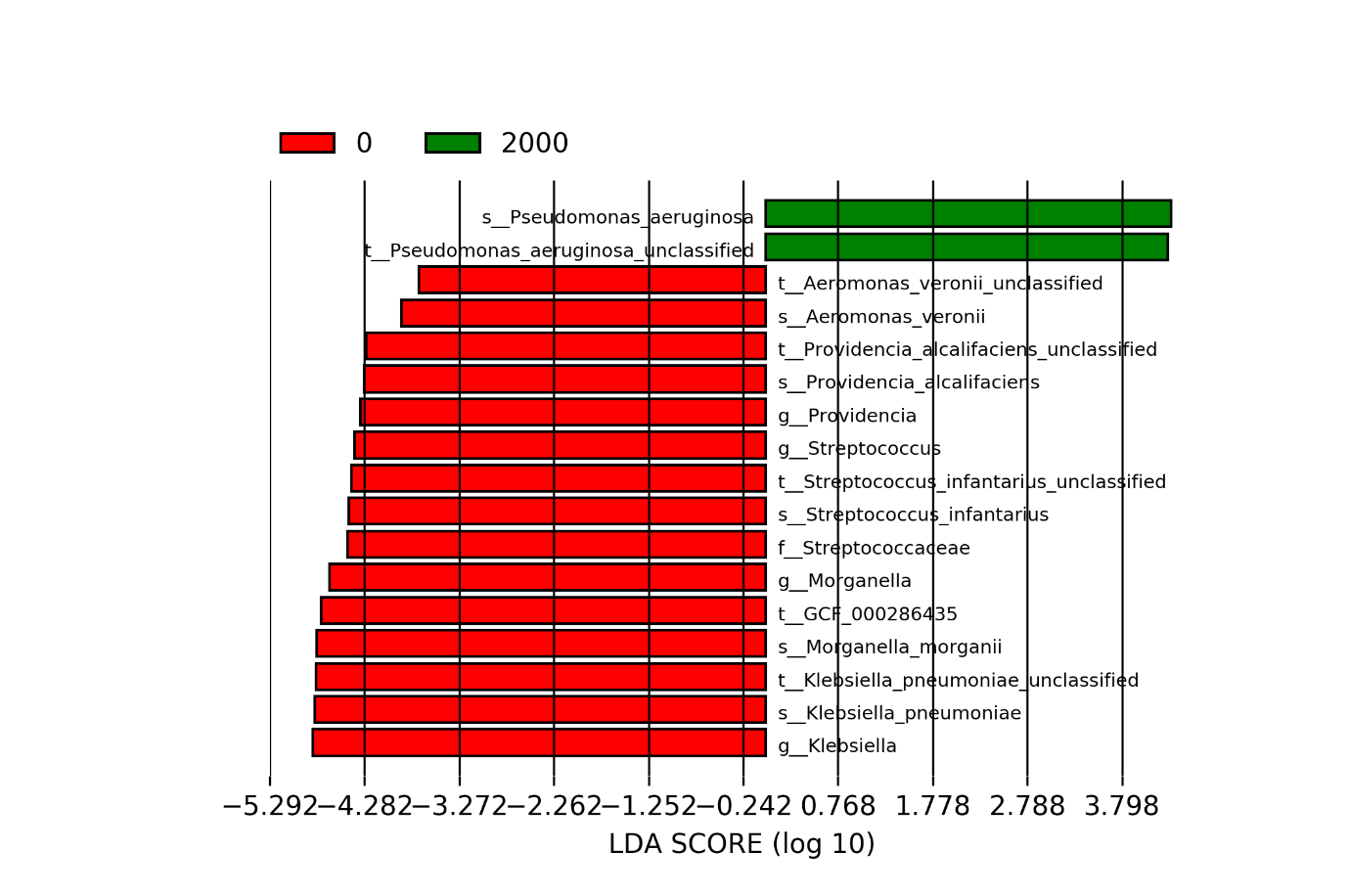


Figure S2. Linear Discriminant Analyses (LDA) effect size (LEfSe) analyses of statistically significant species associated with different cefotaxime treatments. Negative LDA scores (red) show species enriched in the no antibiotic treatment, and positive LDA scores (green) show species enriched in the 2000 µg/L cefotaxime treatment.
