# Supplementary material for: Novel Insights into Selection for Antibiotic Resistance in Complex Microbial Communities
Source: mBio. 2018 Jul 24;9(4):e00969-18. doi: 10.1128/mBio.00969-18 (PMC6058293; doi:10.1128/mBio.00969-18)
Supplement: FIG S3 [file mbo004183973sf3.docx]

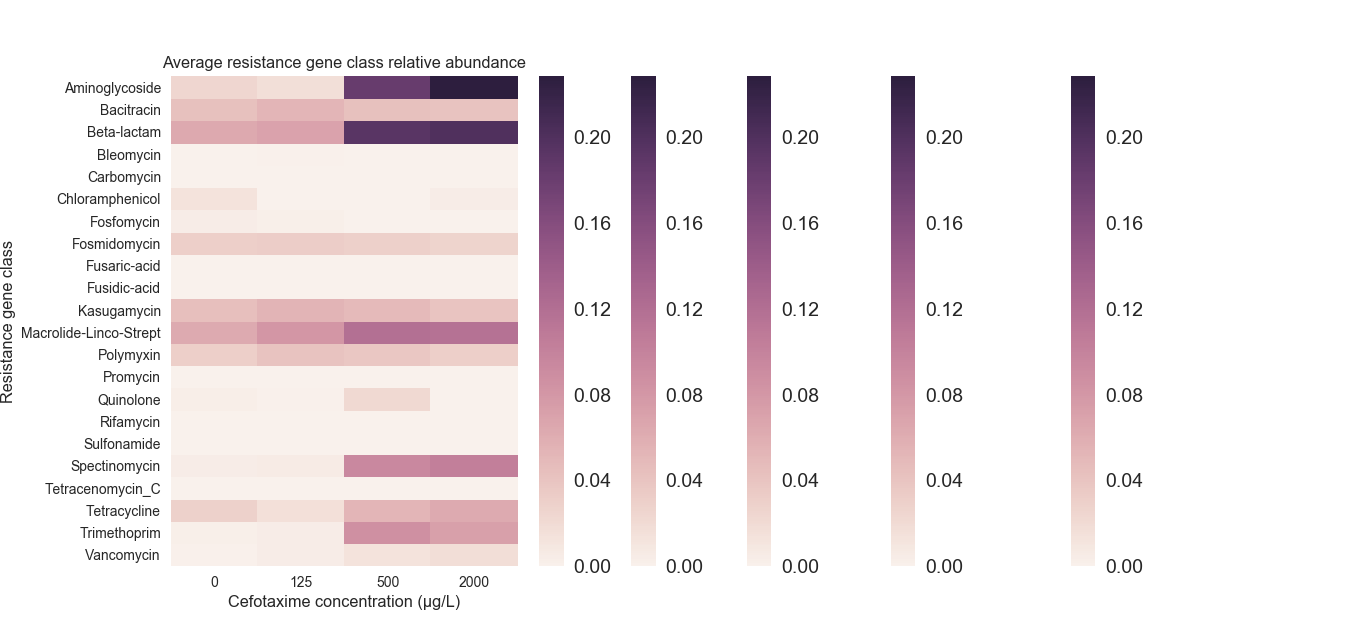


Figure S3. Heatmap showing average (biological replicate n=3) resistance gene relative abundance (resistance gene number normalised with 16S rRNA copy number), following 8 days culture with cefotaxime. “Macrolide-Linco-Strept” = Macrolide, Lincosamide and Streptogramin resistance.
