# Supplementary material for: Novel Insights into Selection for Antibiotic Resistance in Complex Microbial Communities
Source: mBio. 2018 Jul 24;9(4):e00969-18. doi: 10.1128/mBio.00969-18 (PMC6058293; doi:10.1128/mBio.00969-18)
Supplement: FIG S4 [file mbo004183973sf4.docx]

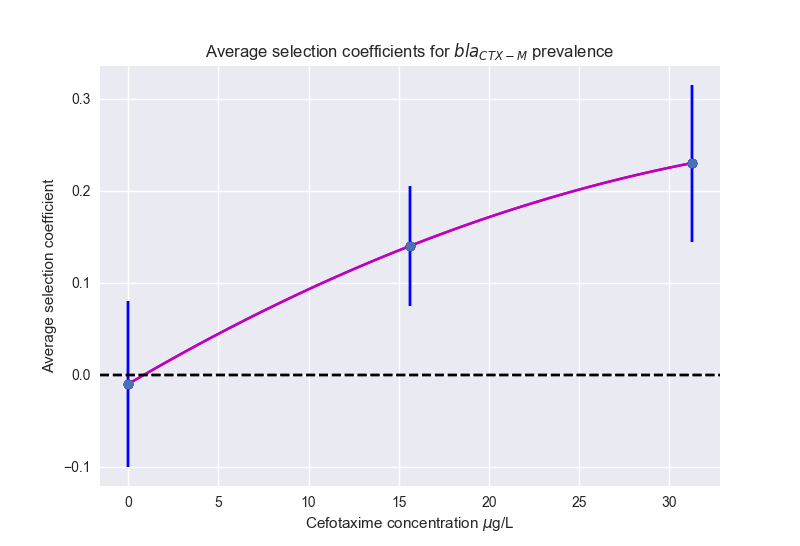


Figure S4. MSC (cefotaxime concentration at the x-axis intercept) determination using average (n=5) selection coefficients (natural log of *bla*_CTX-M_ prevalence over 8 days, *bla*_CTX-M_ prevalence = *bla*_CTX-M_ copy number/16S rRNA copy number, qPCR technical replicate n=2). Shown with standard error bars (of biological replicates) and polynomial (order 2) line of best fit.
