# Supplementary material for: Novel Insights into Selection for Antibiotic Resistance in Complex Microbial Communities
Source: mBio. 2018 Jul 24;9(4):e00969-18. doi: 10.1128/mBio.00969-18 (PMC6058293; doi:10.1128/mBio.00969-18)
Supplement: FIG S5 [file mbo004183973sf5.docx]

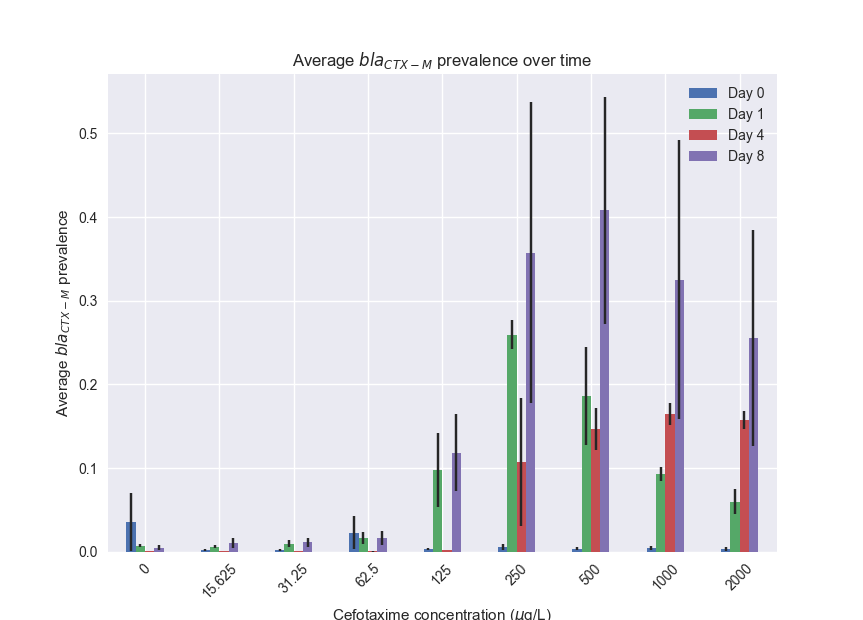


Figure S5. Average (biological replicate n=5, technical qPCR replicate of each biological replicate n=2) bla_CTX-M_ prevalence (bla_CTX-M_ copy number/16S rRNA copy number) number at day 0 and following 1, 4 and 8 days of cefotaxime exposure. Shown with standard error bars (of biological replicates).
