# Supplementary material for: Novel Insights into Selection for Antibiotic Resistance in Complex Microbial Communities
Source: mBio. 2018 Jul 24;9(4):e00969-18. doi: 10.1128/mBio.00969-18 (PMC6058293; doi:10.1128/mBio.00969-18)
Supplement: FIG S6 [file mbo004183973sf6.docx]

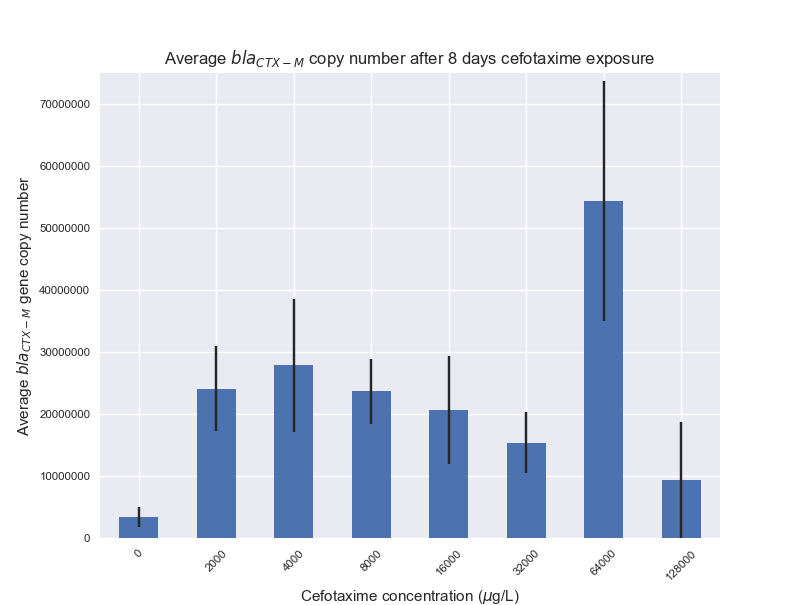


Figure S6. Average (biological replicate n=5, technical qPCR replicate of each biological replicate n=2) bla_CTX-M_ copy number following 8 days cefotaxime exposure in the higher concentration experiment. Shown with standard error bars (of biological replicates).
