# Supplementary material for: Novel Insights into Selection for Antibiotic Resistance in Complex Microbial Communities
Source: mBio. 2018 Jul 24;9(4):e00969-18. doi: 10.1128/mBio.00969-18 (PMC6058293; doi:10.1128/mBio.00969-18)
Supplement: FIG S8 [file mbo004183973sf8.docx]

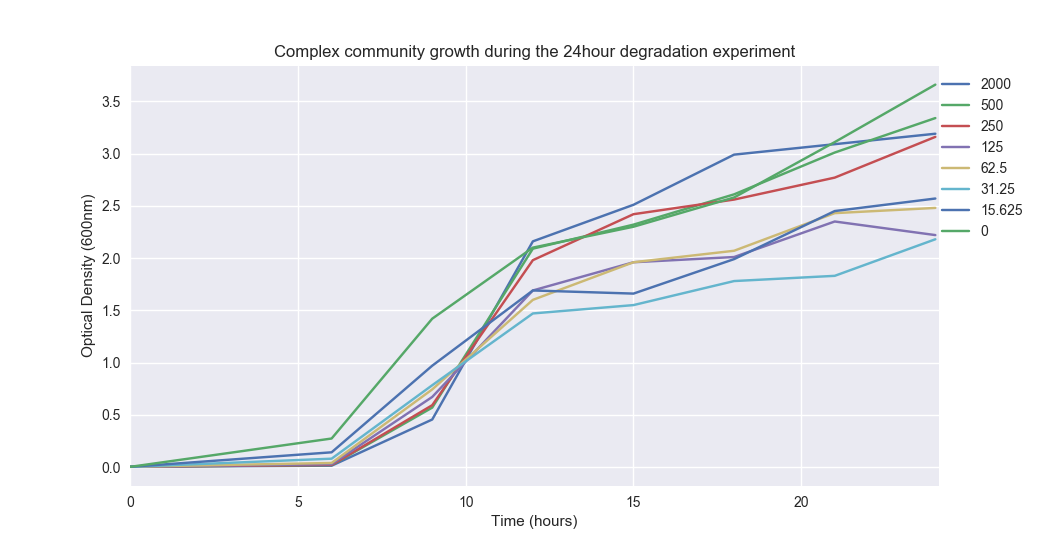
Figure S8. Growth (optical density (600nm)) of the complex community over time during the 24 hour degradation experiment. Single replicate only.
