# Supplementary material for: Novel Insights into Selection for Antibiotic Resistance in Complex Microbial Communities
Source: mBio. 2018 Jul 24;9(4):e00969-18. doi: 10.1128/mBio.00969-18 (PMC6058293; doi:10.1128/mBio.00969-18)
Supplement: TABLE S1 [file mbo004183973st1.docx]

Table S1. Nominal (expected) and average (biological replicate n=3, technical replicate of each n=2) measured cefotaxime concentrations as determined by LC-MS at the beginning (time 0) of the selection experiment, and after 24 hours culture at 180rpm, 37°C in the presence of the complex community. Also shown are the cefotaxime stocks (‘1’ and ‘2’) used in the experiment.

| Nominal Concentration (µg/L) | Measured concentration (µg/L) at time 0 | Measured concentration after 24hours (µg/L) |
| --- | --- | --- |
| 15.625 | 0 | 0 |
| 31.25 | 25.5 | 0 |
| 62.5 | 26.75 | 6 |
| 125 | 46.25 | 0 |
| 250 | 205 | 0 |
| 500 | 438 | 2.6 |
| 1000 | 830 | 0 |
| 2000 | 1686 | 4 |
|  |  |  |
| Stock 1 = 1250 | 1272 | - |
| Stock 2 = 78.125 | 51.56 | - |
